# Science that "knows" and science that "asks"

**DOI:** 10.1186/1479-5876-9-128

**Published:** 2011-08-02

**Authors:** Pierre R Smeesters, Marie Deghorain, Andrew C Steer

**Affiliations:** 1 Laboratory of Bacterial Genetics and Physiology, IBMM, Faculté des Sciences, Université Libre de Bruxelles, Belgium; 2 Centre for International Child Health, Royal Children's Hospital, University of Melbourne, Melbourne, Australia

## Abstract

Clinician-researchers and experimental scientists do not speak the same language; they have different professional environments and different end-points in their research. This creates considerable problems of comprehension and communication, which constitute a major drawback in multidisciplinary work such as translational medicine. A stereotypic representation of both these worlds is presented as a starting point to encourage debate on this issue.

## Introduction

'To doubt everything or to believe everything are two equally convenient solutions: both dispense with the necessity of reflection.' Henri Poincaré

Recent progress in biomedical sciences and technology, such as advances in (meta)genomics, molecular biology and bioinformatics, have radically transformed biomedical research such that multidisciplinary collaborations are often needed [1]. At the same time, multidisciplinary and translational research has become a global research priority and is preferentially considered by many funding agencies [1]. Translation of basic scientific progress into clinical output is certainly an excellent objective. For this translation to be realised as well-funded scientific projects [2-5], there is a well identified need to improve the communication and the relationship between basic experimental scientists (mostly PhDs) and clinicians (MDs) [6]. However, the education and daily life of these two actors have not really changed over time and continue to be driven by different pressures. In the next two paragraphs, a stereotypic representation of both the clinician-researchers and experimental scientists will be presented as exaggerated examples in order to stimulate discussion.

Physicians face a long, drawn out and difficult training program which prepares them to eventually become experienced and wise clinicians. They learn to apply the latest interpretations of scientific data to the benefit of their patients. If the rigor of training has not exhausted them, then clinical evaluation and patient well-being become their focus as they face the human condition with all its magnificence and weakness. Because of the nature of their work, clinicians have an incredible opportunity to share ideas with people from a broad range of socio-economic backgrounds with divergent points of view. These exchanges frequently occur in the setting of acute medical conditions that favour real and honest communication. Clinicians therefore often develop a solid understanding of where societal expectations and moral attitudes towards medical care lie. With time, they become more and more convinced that quick and often lifesaving answers are probably more important than questions. Clinicians are often overwhelmed with patient care, student teaching and administrative tasks. There is precious little time for medical research, nor is there encouragement from employers to do research in the modern health care environment where cost minimisation often drives administrative decision making. After having eaten dry bread during initial years of practice, the clinicians become financially comfortable. As drug prescribers, clinicians are very attractive targets for pharmaceutical marketing. Pharmaceutical companies invest in clinical studies led by successful physicians. The compensation for these leaders sometimes includes generous consultation fees, business class travel or accommodation in five star hotels.

The experimental researchers have to pass through an uncertain and stressful training period. Their supervisors' and their own expectations are very high. They are trained to be critical thinkers, to work on the unknown, sometimes in a solitary environment, to learn from errors, to deal with unexpected experimental problems, and to remain up to date with the literature on their favoured subject. Experimental researchers develop an accurate and cutting edge skill to conduct difficult research projects in the long term. However, they often restrict themselves in a very narrow environment to a specific research topic. Imagination is a must; good questions that can be answered may be pursued for many nights. While dreaming of answers they spend much more time on hypotheses as each answer is a novel question. They also spend a lot of time writing and rewriting research proposals. If their enthusiasm survives, they are faced with cut-throat competition for honours and funding. They might still live on short-term grants even after the second post-doctoral tenure. Pharmaceutical companies do not court them. They wonder where the stars of the hotels are while chewing a two-dollar dry cheesecake from the canteen at the congress venue.

## Discussion

Of course, reality is much more intricate and subtle. The notion of the conflict of interest, for example, has received significant attention for some time [7]. Significant and progressive changes have since been made in public and publishing policies to minimise this as a potential issue. However, real differences still exist between the experimental scientist and the clinician which affect communication between the two groups. As noted by Philip Watanabe: 'attempting to organize symposia where experimental researchers and clinicians truly interact for value to advances in medicine is difficult, if not impossible.' [8]. Clinicians and experimental scientists do not speak the same language; they have different professional environments and different end-points in their research [9]. Basic experimental science might be seen as asking much more than it answers while clinical research often focuses on pragmatic answers. Basic scientists sometimes regard clinical research as not quite respectable, at least at a scientific level. They naturally do not feel comfortable with clinical situations and clinicians may not help them in being so. If MD-trained or young PhDs scientists try to cross the bridge between the disciplines, they often face incomprehension. Those scientists 'can be seen as second class researchers if they are not elucidating the latest of mechanisms for the basic sciences' [8]. Declan Butler characterised this chasm between basic science and clinical practice in a special issue of Nature by using the title: 'Crossing the valley of death' [1].

The position of physician-scientist has emerged as a potential solution to these problems. Numerous medical schools started MD-PhD programs in the mid 20^th ^Century and this has been supported by the NIH [10]. Physician-scientists may be the catalysts of translational research [11] because they represent a crucial link in the chain of scientific discovery [12]. To maintain this role, they need to share their time, energy and financial resources between the practice of medicine and the conduct of research. Several Nobel prize winners including Michael Brown (1985) and Sir James Black (1988) testify to the potential successes of this kind of research career [11]. However, does the exponential increase in our understanding of human pathophysiology, in the complexity of modern clinical care and in the technical capability of experimental techniques still allow individuals to be proficient in clinical medicine and basic science simultaneously? Andrew Schafer underlines that 'the vast and dramatically changing bodies of knowledge in these arenas of medicine have made it humanly impossible for any one individual to attain even a semblance of mastery of much of it' [6]. Declan Butler even believes that 'science and innovation have become too complex for any nostalgic return to the physician-scientist on their own as the motor of health research' [1]. Does the physician-scientist need to make a choice between medical practice and research at some point of his/her career [9]? The question is open and the answer may vary at different stages of his/her career development. It has been asked during the three last decades whether physician-scientists might be an 'endangered' [13-15] or a 'vanishing' [6] species. The number of candidates for such a career is apparently decreasing and success in grant applications by physician-scientists has dropped substantially in the last decade [6]. Well-trained physician-scientists are however still in high demand in the private and academic sector [16,17]. While training more physician-scientists may be one part of the solution to this global problem, it will not on its own bridge the gap between practising clinicians and experimental scientists.

If the physician-scientist is a vehicle for exposing a physician to the rigor of basic science, why does the corollary not frequently exist? Why is a hospital so closed to non 'clinical care' professionals? It is rare for basic scientists to become involved in professional activities outside the restricted and homogeneous universe of their laboratory. At the very least, cross-departmental research projects should be more clearly encouraged and supported. Increased exposure of basic scientists to the clinical coalface could help broaden their view of research opportunities and may produce sparks to fire novel scientific creativity. Aaron Salzberg writes: 'The scientist must not only develop and maintain technological expertise, but must also assure the public that science is being developed and presented in a manner consistent with societal goals' [18]. To achieve this, basic scientists should have both the desire and more opportunity to be immersed into the medical reality.

## Conclusion

The specific expertise and know-how of each actor of biomedicine is of course necessary and essential. However, on well-defined translational projects, a real association of basic-science scientists with research clinicians could be extremely valuable. If this association was to take place on equal footing, it could potentially increase the delivery of societally responsible output. For every such successful association the recipe must include the ingredients of humility, good communication, and an ability to learn, understand and appreciate the other partner's point of view and training background. By mixing the science that "asks" with the science that "knows", we could even produce science that "serves".

## Competing interests

PRS has received a research grant and a congress attendance reimbursement from Pfizer. ACS has received a congress attendance reimbursement from Pfizer and a consultancy fee from Wyeth. MD has no conflict of interest.

## Authors' contributions

PRS is a paediatrician working full time in a basic scientific laboratory for 6 years. His research interest is translational research in microbiology and infectious diseases. MD is a basic science microbiologist working on the gram-positive cell wall organisation and on the characterisation of the bacterial toxin-antitoxin systems. ACS is a paediatrician/paediatric infectious diseases physician and research fellow who is trying to juggle clinical paediatrics with clinical and public health research in the field of group A streptococcal disease. PRS drafted the first version of the MS while MD and ACS significantly improved it. All authors approved the final version.
